# Organizational skills training for children with ADHD: study protocol for a randomized, controlled trial

**DOI:** 10.1186/s13063-021-05499-9

**Published:** 2021-10-29

**Authors:** Aida Bikic, Søren Dalsgaard, Kristoffer Dalsgaard Olsen, Denis G. Sukhodolsky

**Affiliations:** 1 Child and Adolescent Mental Health Services Southern Jutland, Kresten Phillipsens Vej 15, Aabenraa, Region of Southern Denmark Denmark; 2 Department of Regional Health Research, Faculty of Health Sciences, University of Southern Denmark, Winsløwparken 19, 3, Odense C, Denmark; 3 National Centre of Register-based research, Department of Economics and Business, Aarhus University, Fuglesangs Allé 4, Aarhus, Denmark; 4 Child Study Centre, Yale University School of Medicine, 230 South Frontage Road, New Haven, CT USA

**Keywords:** ADHD, Organizational skills training, Organizational difficulties, Executive functions

## Abstract

**Background:**

Problems with sustained attention, impulsivity, and hyperactivity are the most prominent symptoms of attention-deficit hyperactivity disorder (ADHD), but many children with this diagnosis also present with poor organizational skills that are important in relation to school. These problems tend to increase from childhood to adolescence and are often not well managed by medication. Organizational skills training (OST) is a range of behavioral interventions that specifically target organizational skills deficits. Evidence supports the effect of OST on improving organizational skills, inattention, and academic performance in children with ADHD. Because previous clinical trials included mostly children above the age of 8 years, this trial includes children in the age range 6–13 years to expand the knowledge on the effects of OST in younger children. Previous OST research has also shown improvement on inattention in parent ratings; we will investigate if a change in inattention can be confirmed with neurocognitive tests. Finally, little is known about predictors of treatment response in OST.

**Objectives:**

The primary objective is to investigate if OST has positive effects on organizational skills in children with ADHD. The primary outcome measurement is the parent-rated Children’s Organizational Skills Scale (COSS), collected before and at the end of the 10 week intervention. Secondary and exploratory outcomes include inattention ratings, family and school functioning, and cognitive functions measured before the intervention period, immediately after, and at a 6 month follow-up. Additional objectives are to investigate both neurocognitive outcomes and age as predictors of treatment response.

**Methods:**

This is a randomized clinical superiority trial investigating the effect of OST vs a treatment-as-usual (TAU) control group for children with ADHD in the age range of 6–13 years. All participants (*n* = 142) receive TAU. OST is administered in a group format of 10 weekly sessions. Adverse events are monitored by study clinicians during weekly therapy sessions and all assessments. Data analyses will be conducted using mixed linear regression models with random intercepts for patients, adjusted for the stratification variables and the baseline value.

**Perspectives:**

This study will provide important new knowledge and expand on existing research in the field of non-pharmacological treatment of children with ADHD. OST can potentially have a significant impact on the lives of children with ADHD by helping them learn how to cope with their present deficits and to become more independent and self-reliant. It is also important to investigate predictors of treatment response in order to optimize OST.

**Trial registration:**

ClinicalTrials.gov NCT03160378. Registered on May 19, 2017.

**Supplementary Information:**

The online version contains supplementary material available at 10.1186/s13063-021-05499-9.

## Introduction

Attention deficit/hyperactivity deficit disorder (ADHD) is a prevalent neurodevelopmental disorder in childhood [1–3]. An ADHD diagnosis is associated with a number of negative consequences later in life as higher risk of school drop-out [4, 5], a higher susceptibility to other psychiatric disorders, substance abuse, criminality, adverse health events, and premature death [6–11]*.*

ADHD symptoms involve problems with inattention, impulsivity, hyperactivity, and very often a range of cognitive deficits. So far, an ADHD specific cognitive profile has not be identified, as individuals with ADHD present a range of heterogeneous cognitive deficits which are already visible at young age and often stabilize over time [12]. Organizational skills deficits refer to issues such as losing things/forgetfulness, having difficulties with planning or organizing tasks and activities, failing to follow through with instructions, and finishing schoolwork. These four problems with organization and planning ahead are included in the description of ADHD in the Diagnostic and Statistical manual of mental disorders (DSM-5) [13]. Organizational skills deficits are partially an expression of executive dysfunctions that many children with ADHD experience [14–16].

Organizational skills interventions for children and adolescents with ADHD target organizational deficits and include skills for organizing materials (e.g., school notes and handouts) and time (e.g., long-term planning and scheduling) [17]. In ADHD, issues with misplacing materials, procrastination, failure to plan, or organize and prioritize actions all increase in severity over time [18, 19], and often persist into adulthood [20] as the complexity of academic responsibilities increases, while in typically developing individuals such difficulties decrease over time due to brain maturation [21]. Having difficulties with organizational skills often imparts a range of adverse consequences in everyday life including academic underperformance (even in gifted students) and psychosocial, occupational, or economic difficulties [4, 22–26]. In addition, medical treatment for ADHD has limited effect on organizational skills difficulties [27, 28].

Executive functions are the foundations of organizational skills. There is evidence that the development of executive functions starts as early as in pre-school and kindergarten ages and is important for school-readiness and social-emotional development [29]. As these challenges increase with age, it is critical to intervene and address these impairments as early as possible.

Organizational skills interventions include different behavioral techniques like prompting, rehearsal, and contingency management. Parents often play an active part in the treatment by prompting, praising, and rewarding the child with the aim to reinforce specific behaviors and to promote skills generalization. There is a range of different organizational skills interventions, with some interventions focusing strictly on materials organization and others on the process of completing homework and/or parent-child and parent-school relationships surrounding homework [30–33]. Most organizational skills interventions are aiming to teach parents how to monitor, prompt, and reward their child in relation to school related tasks. The ultimate goal is to reduce the frequency of monitoring and overt rewarding over time, helping the child become more independent.

We conducted the first meta-analysis of organizational skills interventions, which included 12 randomized controlled trials (RCT) and 1054 children (576 treatment, 478 control). The results show that the weighted mean effect sizes for teacher-rated organizational skills are moderate and large for parent-rated organizational skills [34]. We also found that organizational skills training had a small to moderate effect on teacher- and parent-rated symptoms of inattention. Additionally, we found small to medium effect sizes in academic performance as rated by teachers [34]. Findings from two previous qualitative reviews also support these results [17, 35].

Organizational skills training is still a developing research area with several unanswered questions. The body of evidence indicates that executive function interventions are working especially well in younger children [36]. Because children in Denmark start school at age 6, we consider inclusion of this age group important. Thus, we are targeting children 6 years of age and older to expand the knowledge on the effects of OST in younger age groups. Additionally, a number of studies have found that inattention as measured by rating scales improved following OST treatment [34]. This trial will investigate if possible improvements in parent-ratings of attention and organizational skills after OST are also paralleled by changes on neurocognitive tests, which has not been addressed in prior studies. Furthermore, neurocognitive tests will also be used to investigate treatment response as it is unclear if OST works better for some individuals than others. Finally, to the best of our knowledge, this is the first randomized and controlled trial of an organizational skills intervention in a European context (specifically in Denmark). It is important to investigate OST in other contexts outside North America, as school systems and requirements vary across countries. The trial will compare OST treatment to treatment-as-usual (TAU) in children with ADHD referred to clinical services. TAU is chosen as the comparator as children with ADHD who are already in treatment are the target of the intervention. We would like to investigate if OST can provide additional benefits for those children

## Methods

### Objectives

The primary objective of this parallel group, superiority trial is to investigate whether organizational skills training has a positive effect on parent-rated organizational skills in 6–13 year old children with ADHD. The secondary objective is to investigate possible effects on teacher-rated organizational skills, parent-rated inattention, objectively measured sustained attention, and family and school functioning. All these measures will be also investigated at baseline, after the intervention, and at a 6 month follow-up. Additionally, we will investigate predictors of treatment response.

### Trial sites

Participants are recruited at two sites in Southern Denmark: Child Psychiatric Department (CPD) Aabenraa and CPD Vejle.

### Consent to participate

The patients’ treatment-as-usual will not be affected by their participation in this trial. Participants are not prohibited to participate in other treatments. The enrolled families can leave the trial without further explanation at any time. Study investigators can exit a subject from the study if subjects require a higher level of psychiatric care (e.g., inpatient hospitalization). To promote participant retention, trial participants will receive three gift cards worth a total of DKK 400. If participants decide to leave the trial earlier, they will receive gift cards for attended assessments. Transportation costs will be reimbursed for all meetings.

In the intervention group families have the option to use vTime, an online timer app. Access to vTime (http://vtime.dk/) is provided free of charge by the company. vTime has no involvement in the trial design, funding, or any other aspect apart from providing access to the online service.

Non-serious adverse events and serious adverse events will be monitored during intervention and at all endpoint assessments and reported to the ethical committee annually. At all assessments, participants are asked about adverse events by the psychologist conducting the outcome assessments. All adverse event logs are reviewed by the principal investigator. The principal investigator will report severe and serious adverse events to the institutional review board. There are no trial steering committees or data monitoring committees involved as this is not required in trials testing low-risk behavioral interventions [37, 38]. Also, external auditing is only required in pharmaceutical trials according to Danish law. We perform internal reviews that are independent from the sponsors on a regular basis, where we check that all the protocol procedures are followed at both sites and data is stored appropriately.

A child can only participate in the trial if the written consent of all legal guardians (referred to as parents throughout the text) is obtained. Danish law requires written consent by the child above the age of 15 years, and as the children in our trial are younger, we do not collect it. All participating children gave assent for participation. Patients will continue their general assessment and treatment procedure. The treating clinicians make the first approach to the family and provide recruitment and information letters to eligible families. If parents choose to contact the project staff, they will be offered an individual information meeting by a clinical psychologist associated with the trial where the trial will be described and discussed in detail. Parents can choose to take an accompanying person to the information meeting for support. After the meeting, families can decide whether to participate in the trial by providing informed consent. Informed consent is collected by a psychologist, who is a trial research assistant.

### Assessments of eligibility

Children in the targeted age group who are currently referred to and/ or already in treatment for ADHD or under assessment for ADHD at one of the two Child Psychiatric Departments are invited to participate in the trial. The diagnostic assessment includes a clinical interview with the child and one or both of the parents using the Kiddie-Schedule for Affective Disorders and Schizophrenia (K-SADS) [39]. K-SADS is a widely used semi-structured clinical diagnostic interview for parents and children. Additionally, all children are assessed with Reynolds Intellectual Assessment Scales (RIAS) [40] and Behavior Rating Inventory of Executive Functions (BRIEF) (parent edition) [41]. Finally, children are included in the trial if they comply with the following inclusion and exclusion criteria:

### Inclusion criteria

The following criteria are required for inclusion: ADHD diagnosis according to K-SADS, age between 6 and 13 years (both inclusive), IQ above 80, and written informed consent. Additionally at least one standard deviation (SD) above the mean on the Plan/Organize subscale on the BRIEF verifying problems with organizational skills is required. Both children with and without pharmacological treatment are included in the trial, although we expect the majority of children to receive pharmacological treatment. Children are required to be on stable pharmacological treatment for at least 6 weeks before participation in the trial. Families are recommended not to change the child’s ADHD medication or initiate new medications during the trial period of 10 weeks; however, the final decision about medication is made by the parents and the treating specialist, who is unrelated to the trial. The study researcher team will inquire about possible changes in medication at assessment visits and all changes will be summarized and reported in the results paper.

### Exclusion criteria

**Fig. 1:**
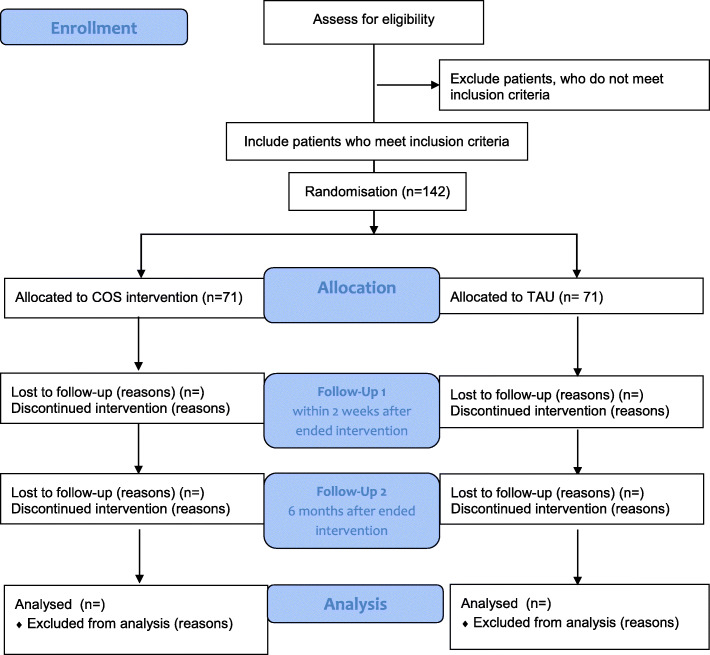
CONSORT flow diagram. Flowchart on participant enrollment and the randomization process

Patients fulfilling any of the following criteria as assessed by the K-SADS are excluded: autism spectrum disorders, serious psychopathology requiring immediate clinical attention (e.g., severe depression or aggressive behavior), head injury or verified neurological disease, intelligence quotient (IQ) < 80, medical condition requiring primary treatment, or no informed consent from parents (Fig. 1).

### Intervention

Children and their parents allocated to the intervention and control group will both receive treatment-as-usual (TAU) at the clinic, and this may include psychoeducation, counseling of parents and teachers at school, and for some children pharmacological treatment. TAU is very similar at both sites and will be recorded.

The intervention group will participate in manualized organizational skills training (OST) in groups (Bikic & Sukhodolsky, in preparation). Parents and children from 4 to 5 families participate in ten weekly 1.5 h group sessions at the clinic. Each group is run by two therapists. In the first half of each session, children and parents are together, and in the second half, parents and children are in separate groups. The content of the intervention is adjusted with age-appropriate tasks: one set of tasks for younger children (6–9 years) and one for older children (10–13 years). The intervention targets organizational skills, planning, and time and task management (homework and school assignments).

### Comparator/control condition

Participants randomized to the control condition continue to receive TAU as scheduled during the trial. Participants randomized to TAU will be offered OST after the completion of end-point and 6-month follow-up assessments in order to promote retention. TAU is provided outside of the study and is not under control of the study investigators. TAU at the clinic is involving clinical assessment for ADHD and treatment. Assessment usually consists of a parent and child interview, questionnaires collected from both home and school and often a school observation. Additionally, the child undergoes cognitive and IQ tests. TAU for children with a clinical ADHD diagnosis is involving psychoeducation, counseling of parents and school, and possibly medical treatment. No concomitant treatments are specifically prohibited, but families are asked not to initiate new behavioral or pharmacological interventions for the duration of the intervention period of 10 weeks. Concomitant treatments are documented for each participant for the duration of the study and will be reported in the participants’ characteristic section of the results paper.

### Randomization

Every participant is assigned a participant number. Participants are randomized 1:1 to intervention or control treatment and stratified by trial site (“Aabenraa,” “Vejle”) and age (older/younger). The allocation sequence is computer-generated with a varying block size for which the investigators are blinded. Randomization is performed by a statistician unrelated to the trial. The research assistant (RA) who is carrying out allocation will have no access to allocation sequence. When the subject is ready to be randomized, the RA will send this subject’s ID (and information relevant to stratification) to the statistician. The statistician will enter the ID number in the web-based randomization algorithms and will inform the RA to which study condition the subject is allocated to. The RA will then inform the families about the allocation and enroll them in the trial.

### Blinding

**Fig. 2:**
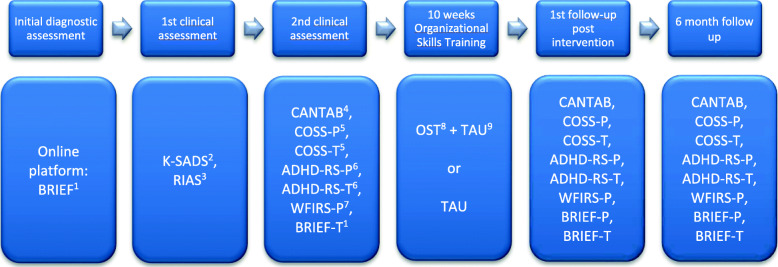
Timeline of assessment phases in the trial. (1) BRIEF, Behavior Rating Inventory of Executive Functions. P, parent. T, teacher; (2) K-SADS, Kiddie-Schedule for Affective Disorders and Schizophrenia; (3) RIAS, Reynolds Intellectual Assessment Scales; (4) CANTAB, Cambridge Automated Neurocognitive Test Battery; (5) COSS, Children’s Organizational Skills Scales. P, parent. T, teacher; (6) ADHD-RS, Attention Deficit Hyperactivity Disorder-Rating Scale. P, parent. T, teacher; (7) WFIRS-P, Weiss scale of disability Parent Report; (8) OST, Organizational Skills Training TAU; and (9) treatment as usual

As the current trial compares an intervention to TAU, it is not possible to blind the participants and their parents. Blinding will be employed in all other possible areas (e.g., administration of the cognitive tasks is carried out by blinded research assistants, randomization, and data analysis) (Fig. 2).

### General description of outcome measures

The following outcome measures are used in this trial:

*Children Organizational Skills Scale (COSS)*—a valid and reliable questionnaire [42]. COSS is a 4-point scale ranging from “hardly ever/never” to “just about all the time” and has 58 items for parents and 35 items for teachers. It has excellent internal consistency alphas for both parent and teacher items, αs = .98 and .97, respectively, and test/retest reliability *r* = .99 and .94, respectively.

*Behavior Rating Inventory of Executive Function (BRIEF)* [43] is an 86-item questionnaire for parents and teachers regarding different aspects of the child’s executive functions. It consists of eight clinical scales and two validity scales. BRIEF has shown high internal consistency (αs = .80–.98) and test/retest reliability (*r*s = .82 for parents, .88 for teachers).

*Cambridge Neuropsychological Test Automated Battery* (CANTAB) is a battery of digital cognitive tests. It consists of highly sensitive, precise, and objective measures of cognitive function [44]. In this trial, we are using the following tests: (a) a test of sustained attention (Rapid Visual Information Processing (RVP), (b) an executive functions measure (One touch Stockings of Cambridge (SOC), (c) a working memory measure (CANTAB Working Memory), (d) a measure of impulsivity (CANTAB Stop Signal Task (SST)), and (e) a measure of reaction time (CANTAB Reaction time task (RTI).

*ADHD-RS* is a 3 -point Likert scale for parents and teachers [45], and it consists of an inattention subscale (questions 1–9), a hyperactivity/impulsivity subscale (questions 10–19), and the Danish version of ADHD-RS also includes a conduct disorder subscale (questions 20–26).

*Weiss Functional Impairment Rating Scale (WFIRS)* is the only measure of functional impairment that looks at specific domains of functioning and has been validated in the ADHD population [46]. WFIRS is a 50-item Likert scale ranging from 0 (never or not at all) to 3 (very often or very much) and is targeting 6 different domains. WFIRS is psychometrically validated with an internal consistency >.8 for each domain and for the scale as a whole.

For an overview of all outcomes and assessments, please see Table 1.

**Table 1:**
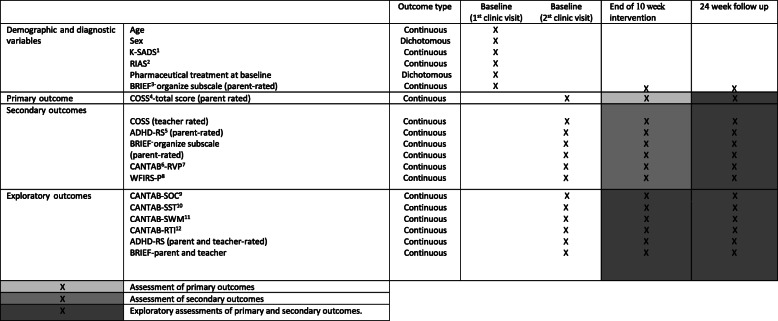
Outcomes and time points for assessment in the OST trial

^1^Kiddie-Schedule for Affective Disorders and Schizophrenia (K-SADS)

^2^Reynolds Intellectual Assessment Scales (RIAS)

^3^Behavior Rating Inventory of Executive Functions (BRIEF)

^4^Children Organizational Skills Scale (COSS)

^5^Attention Attention Deficit-Hyperactivity Deficit-Rating Scale (ADHD-RS)

^6^Cambridge Automated Neurocognitive Test Battery (CANTAB)

^7^Rapid Visual Information Processing (RVP); RS)

^8^Weiss scale of disability-Parent Report (WFIRS-P)

^9^Sockings of Cambridge (SOC)

^10^Stop Signal Task (SST)

^11^Spatial working memory (SWM)

^12^Reaction time task (RTI)

### Outcomes

#### Primary outcome

The primary outcome is the total score on the parent-rated behavior Children Organizational Skills Scale (COSS)-parent [42]. The COSS was translated into Danish following standard procedure for translation of questionnaires; it was first translated from English into Danish by an authorized translator and then back-translated into English by another authorized translator. The final translation was approved by the original developer and publisher.

#### Secondary outcomes

The following secondary outcomes will be assessed at baseline and the end of the intervention.

- CANTAB Rapid Visual Information Processing (RVP)
- Children Organizational Skills Scale (COSS) (teacher edition) [42]
- Inattention subscale ADHD-Rating Scale (ADHD-RS-IV) (parent edition) [45].
- Behavior Rating Inventory of Executive Functions (BRIEF) organize subscale (parent edition) [41].
- Weiss scale of disability-Parent Report (WFIRS-P) [46]

#### Exploratory outcomes

The following exploratory outcomes will be assessed at baseline and at the end of the intervention:

- BRIEF (parent and teacher-edition)
- Inattention subscale assessed by ADHD-RS-IV (teacher edition)
- ADHD-RS-IV (parent and teacher): hyperactivity, ODD subscale and total score
- CANTAB one touch Stockings of Cambridge (SOC)
- CANTAB Working Memory (WM)
- CANTAB Stop Signal Task (SST)
- CANTAB Reaction time task (RTI)

Additionally, all outcomes will be assessed again at the 6 month follow-up.

### Statistical plan and data analysis

#### Sample size

We are conducting a randomized controlled trial using the continuous response variable COSS-total parent rating from independent control and experimental participants allocated at a 1:1 ratio. The primary outcome measure COSS has been tested in previous trials [30, 31].

Using data from Pfiffner and colleagues [31], we conducted a sample size calculation based on the COSS total score by parent rating as primary outcome. Based on a minimal relevant difference of 0.30 and a standard deviation of 0.36, it requires 32 participants in each group to reach 90% power with 5% significance level. Furthermore, to have sufficient power to analyze secondary outcomes with standardized effect size 0.5 SD, the required sample size was doubled to *n* = 64 in each group, resulting in 80% power for the secondary outcomes using 5% significance level. Finally, to account for an expected dropout of 10% during the study period, the required sample size was increased to 71 in each group (total sample size *n* = 142).

#### Analytical model

Descriptive analyses will be conducted of patient characteristics and patient compliance issues (e.g., how many sessions each family has been attending). Since repeated measures of data will be collected for each participant, the primary and secondary outcomes will be analyzed as mean change from baseline to post-intervention using mixed linear regression models with random intercepts for patients, adjusted for the stratification variables (site and age) and the baseline value of the outcome variable. Medication status will be used as a covariate in data analysis. Furthermore, for the primary and secondary outcomes, mixed linear regression models will be used to perform subgroup analyses according to age. The participants will be divided into two age groups of children (6 to 9 years old and 10 to 13 years old). We will perform a test of interaction to assess whether the effect of the intervention is different among the younger children compared with the older children. If the *p* value of the test of interaction is < 0.05, we will present separate estimates for the two subgroups. Finally, exploratory per-protocol analyses of the primary and secondary outcomes will be conducted. In case of variance inhomogeneity or non-normality of the model residuals, cluster robust standard errors will be used. Multiple imputation methods will be applied in case of unbalanced missing data patterns in the control and experimental groups. All analyses will be conducted in Stata 16.

#### Monitoring of patient compliance issues

We are recording how many sessions each family has been attending. The OST group therapists are conducting treatment fidelity checklists after each session and register how well the parents and the children are participating in the sessions and if all of the aims for the session have been reached. Parents are asked to do some tasks with their child at home between sessions (e.g., making and implementing a reward chart). The homework is being reviewed in the following sessions and it is an item on the fidelity checklist. Additionally, at the end of the 10-week intervention, participating parents are asked to fill out a satisfaction survey regarding the intervention.

#### Treatment fidelity

All parent group therapists are trained by the principal investigator (AB) in the use of the OST manual. This training includes conducting the full version of the OST training with an experienced therapist and receiving 10 h of supervision. Thus, each therapist receives 25 h of training and supervision before delivering OST group treatment by themselves. While parent group therapists are responsible for the therapeutic content of OST, child group facilitators are mainly engaging with the children in different structured play activities and receive 5 h of training. After each session, OST group therapists and child group facilitators are filling out a treatment fidelity checklist regarding participant engagement and session treatment aims. Fidelity checklists are used to ensure that the OST sessions are conducted according to the manual and that the program for each session has been addressed by the therapist/facilitator. Treatment during the study is not modified based on treatment fidelity ratings. Possible issues with adherence to the intervention are discussed and addressed in weekly meetings with the principal investigator.

#### Data storage

All participant information is collected electronically at baseline and endpoints. Our questionnaires are located in the REDCap database, which is a secure web application compliant with the General Data Protection Regulation (GDPR) where questionnaire data is automatically stored. The REDcap has a log of data entry and verification. All data is collected using a participant number and is pseudo anonymized. Only study investigators and statisticians will have access to the final data set.

## Discussion

In this trial, we are investigating the effect of a non-pharmacological group intervention for children with ADHD targeting organizational skills in children. This trial is going to fill the gap in existing literature regarding how OST is working for younger children. Because organizational skills deficits are strongly associated with functional outcome, it is important to target interventions to improve these early in life. Although the target of OST is primarily organizational skills, many trials have found that the parents report improvements in their child’s attention. It is unclear if the parent reports indicate a real change or placebo effects, as parents mostly are not blinded to their child’s allocation. By investigating attention with an objective cognitive test, this trial is going to clarify whether OST can improve attention. Additional predictors of treatment response have so far only been investigated by one trial [47] and only in terms of symptom severity, parental anxiety, and IQ. We intend to use objective cognitive measures at baseline to investigate possible predictor effects.

In our meta-analysis of OST studies [34], we found that the most OST studies included children 8 years of age and older, with the exception of Mautone and colleagues [48], who successfully focused on children in kindergarten and first grade. Given that impairments in attention and organizational skill increase in severity with age, and due to the academic demands and decrease in adult supervision, clinical judgment suggests that earlier interventions may promote the acquisition of skills and avert future problems. Behavioral interventions for young children with ADHD are shown to reduce impulsivity and noncompliance [49], but deficits in attention and organization may be overlooked until older ages when compounded by functional impairments. Therefore, we extended the age to 6 years old, as many of the skills in OST are also relevant to younger children, who still need help from their parents. Children older than 13 years of age have different academic tasks and there are other OST interventions for teenagers [50].

Our trial design has several strengths: participants are all referred or in treatment in child and adolescent psychiatric services for ADHD, representing a real clinical sample of children with ADHD. All of our participants score at least one standard deviation above the mean at the plan/organize BRIEF subscale, thereby representing a sample of children with ADHD who have moderate to severe difficulties with organizational skills at baseline, preventing a ceiling effect of the intervention. Organizational skills training is an intervention that might be very feasible and useful in the clinics.

One limitation in our trial is that we are unable to conceal the group allocation from the families which might lead to a certain placebo effect. Additionally, our population group is limited to children with ADHD with possible comorbidities, but autism spectrum disorders (ASD) are excluded. Even though ASD is often comorbid with ADHD, children with ASD may have a wide range of additional treatment needs for the core and co-occurring symptoms other that inattention. OST is a focused treatment for organizational deficits in children with ADHD, and the main aim of our study is to test the efficacy of OST in children with ADHD. While many children with a primary diagnosis of ASD show some degree of ADHD symptoms, only 14% of children with a primary diagnosis of ADHD also have ASD, according to the CDC [51]. Our study is not powered to test moderating effects of co-occurring ASD, and this issue will likely require a separate, adequately powered clinical trial.

## Trial status

Trial registration: ClinicalTrials.gov Identifier: NCT03160378, registered May 19, 2017. https://clinicaltrials.gov/ct2/show/NCT03160378?term=aida+bikic&draw=2&rank=2

Protocol version 8, 29.05.2020. The first participant was enrolled May 22, 2017. Recruitment is currently ongoing till June 2022. It was not possible to submit the manuscript earlier due to recruitment obligations and the main author being on maternity leave for a year. Detailed trial registration data set is presented on Table 2.

**Table 2:** Trial registration data set

Data category	Information^**32**^
Primary registry and trial identifying number	ClinicalTrials.gov
	NCT03160378
Date of registration in primary registry	May 19, 2017
Secondary identifying numbers	S-20160180, ID 17/7467
Source(s) of monetary or material support	Psychiatric Research Foundation in Region of Southern Denmark and Jascha Fonden
Primary sponsor	Psychiatric Research Foundation in Region of Southern Denmark
Secondary sponsor(s)	Jascha Fonden
Contact for public queries	Aida Bikic e-mail: aida.bikic@rsyd.dk
Contact for scientific queries	Aida Bikic e-mail: aida.bikic@rsyd.dk
	University of Southern Denmark, Odense, Denmark
Scientific title	Organizational skills training for children with ADHD
Countries of recruitment	Denmark
Health condition(s) or problem(s) studied	Attention Deficit Hyperactivity Disorder (ADHD)
Intervention(s)	Active comparator: Organizational skills training (OST), a group treatment for families
	Control group: Treatment as usual (TAU)
Key inclusion and exclusion criteria	Ages eligible for study: 6-13 years
	Sexes eligible for study: both
	Accepts healthy volunteers: no
	Inclusion criteria: child age 6-13 years, ADHD diagnosis, organizational skills deficits
	Exclusion criteria: autism spectrum disorders, serious psychopathology requiring immediate clinical attention (e.g., severe depression or aggressive behavior); head injury or verified neurological disease; intelligence quotient (IQ) < 80; medical condition requiring primary treatment, no informed consent from custody.
Study type	Interventional
	Allocation: randomized intervention model. Parallel assignment masking: investigator and outcomes assessor are blinded.
	Primary purpose: treatment
	Phase III
Date of first enrolment	May 22, 2017.
Estimated primary completion date	June 2022
Estimated study completion date	December 2022
Target sample size	142
Recruitment status	Recruiting
Primary outcome(s)	Total score on the Parent-rated behavior Children Organizational Skills Scale (COSS)
Key secondary outcomes	• CANTAB Rapid Visual Information Processing (RVP) • Children Organizational Skills Scale (COSS) (teacher edition)-total • Inattention subscale assessed by ADHD-Rating Scale (ADHD- RS-IV) (parent edition). • Behavior Rating Inventory of Executive Functions (BRIEF) organize subscale (parent edition). • Weiss scale of disability-Parent Report (WFIRS-P)

## Supplementary Information

**Additional file 1.** Review history.

## Data Availability

Not applicable as this is a protocol paper. After the termination of the trial, data will be stored at the Danish National Archives, from where data can be requested.

## Abbreviations

ADHD: Attention deficit hyperactivity disorder
ADHD-RS: Attention Deficit Hyperactivity-Rating Scale
BRIEF: Behavior Rating Inventory of Executive Functions
CANTAB: Cambridge Automated Neurocognitive Test Battery
COSS: Children Organizational Skills Scale
CPD: Child Psychiatric Department
K-SADS: Kiddie-Schedule for Affective Disorders and Schizophrenia
OST: Organizational Skills Training
RIAS: Reynolds Intellectual Assessment Scales
RTI: Reaction time task
RVP: Rapid visual information processing
SWM: Spatial working memory
SOC: Stockings of Cambridge
SST: Stop Signal Task
TAU: Treatment-as-usual
WFIRS-P: Weiss scale of disability-Parent Report
