# Supplementary material for: Organizational skills training for children with ADHD: study protocol for a randomized, controlled trial
Source: Trials. 2021 Oct 29;22:752. doi: 10.1186/s13063-021-05499-9 (PMC8556963; doi:10.1186/s13063-021-05499-9)
Supplement: Supplementary file 1 — Additional file 1. Review history. [file 13063_2021_5499_MOESM1_ESM.zip › 13063_2021_5499_ReviewerReport_V0_R1.pdf]

Reviewer's report

Title: Organizational skills training for children with ADHD Study protocol for a randomized, controlled trial

Version: 0 **Date**: 19 Mar 2021

Reviewer: Charlotte Lucy Hall

Reviewer's report:

This paper presents a protocol for an RCT looking at organisational skills training for children with ADHD. Participants are randomised to receive OST+TAU or just TAU. Although the study is well written and is in an interesting, it does not follow SPIRIT guidelines and thus is missing significant information which makes it hard to judge the credibility of the design and protocol. My subsequent detailed comments reflect this.

Title: randomised controlled trial rather than randomised "and" controlled trial is more typical terminology

Abstract: Does not conform to SPIRIT guidance. There are no details on primary outcome/end point, analysis plan or safety measures.

Introduction: Covers a good range of relevant literature. This does feel a little lengthy at times but comprehensive. I would like to see a justification for the choice of comparator here.

Method:

Pg 5: you don't say who makes the first approach to the family.

Pg 5: you use the term "child custody" but elsewhere only refer to parents. I think you may mean parent/legal guardian? This should be explained when first mentioned and then you can just use one term throughout

Pg 5; who conducts in the information session?

Pg 5: is consent written? Does the child provide assent?

Why is the inclusion 6-13 years? Can this be justified

Pg 7: inclusion criteria - will you account for whether participants are on meds in the analysis? Might this not affect how well they engage with the skills training (i.e. focus/activity) even if their organizational skills are not adequately managed on meds? Will changes to meds count as a protocol deviation? Will these be recorded?

Exclusion criteria - why exclude all ASD when this is highly co-morbid with ADHD? Does this limit the utility of the findings in real world practice.

Pg 8: will you record what TAU is at each site?

Pg 9: Is randomisation via web-system? Who will randomise? You say researchers are blinded to block sizes, but I think they blind to allocation also (this needs to be more clear in randomisation heading).

There is no mention of ethical clearance? Only in the sub-heading at the end. This should be in the text. Also, are there are oversight committees (Trial steering committee or data monitoring committee) who may over see this?

It is not clear who funded the trial in the main text and what their role is in the trial conduct.

There is no mention of auditing.

There's no mention of quality checks on data entry or who will enter the data

In general, the paper requires more detail before being suitable for publication.

Publons Reviewer Recognition. Springer Nature can send verification of this review directly to Publons (a subsidiary of Clarivate Analytics). If you would like to take advantage of this service, please click on the “Yes” option below. Your name, email address, title of the reviewed manuscript, name of the journal, and date of your review submission (the “Review Data”) will then be transmitted to Publons after the final decision on the manuscript has been made. If you have already registered at Publons, they will notify you of the receipt of this review and update your profile as per your settings and their policy. If you are not registered with Publons, you will receive an email from them asking you to register in order for them to be able to recognize your review on your new profile page. Publons may use the Review Data to generate derivative metadata for the benefit of Publons and you as a reviewer, carefully considering the sensitivity of such information. For example, Publons may verify your record as a reviewer by updating your profile published on its webservice if you have registered for such service or help editors to identify candidate reviewers. Please find the details of processing in Publons’ privacy policy https://publons.com/about/terms

Yes

Level of interest

Please indicate how interesting you found the manuscript:

An article whose findings are important to those with closely related research interests

Quality of written English

Please indicate the quality of language in the manuscript:

Acceptable

Quality of figures

All images and figures within the manuscript should be genuine i.e. without evidence of manipulation. No specific feature within an image may be enhanced, obscured, moved, removed, or introduced. If you have concerns about the veracity of the figures you should choose the first option below.

Acceptable

Statistical review

Is it essential that this manuscript is seen by an expert statistician? If so, please give your reasons in your report.

Yes, but I do not feel adequately qualified to assess the statistics

Declaration of competing interests

Please complete a declaration of competing interests, considering the following questions:

1. Have you in the past five years received reimbursements, fees, funding, or salary from an organisation that may in any way gain or lose financially from the publication of this manuscript, either now or in the future?
2. Do you h hold any stocks or shares in an organisation that may in any way gain or lose financially from the publication of this manuscript, either now or in the future?
3. Do you hold or are you currently applying for any patents relating to the content of the manuscript?
4. Have you received reimbursements, fees, funding, or salary from an organization that holds or has applied for patents relating to the content of the manuscript?
5. Do you have any other financial competing interests?
6. Do you have any non-financial competing interests in relation to this paper?

If you can answer no to all of the above, write 'I declare that I have no competing interests' below. If your reply is yes to any, please give details below.

I declare that I have no competing interests

I agree to the open peer review policy of the journal. I understand that my name will be included

on my report to the authors and, if the manuscript is accepted for publication, my named report including any attachments I upload will be posted on the website along with the authors' responses. I agree for my report to be made available under an Open Access Creative Commons CC-BY license (http://creativecommons.org/licenses/by/4.0/). I understand that any comments which I do not wish to be included in my named report can be included as confidential comments to the editors, which will not be published.

I agree to the open peer review policy of the journal

Were you mentored through this peer review?

No
