# Supplementary material for: Organizational skills training for children with ADHD: study protocol for a randomized, controlled trial
Source: Trials. 2021 Oct 29;22:752. doi: 10.1186/s13063-021-05499-9 (PMC8556963; doi:10.1186/s13063-021-05499-9)
Supplement: Supplementary file 1 — Additional file 1. Review history. [file 13063_2021_5499_MOESM1_ESM.zip › 13063_2021_5499_ReviewerReport_V0_R2.pdf]

Reviewer's report

Title: Organizational skills training for children with ADHD Study protocol for a randomized, controlled trial

Version: 0 **Date**: 5 Apr 2021

Reviewer: Alexander Gough, MA VetMB

Reviewer's report:

Thanks for this interesting and useful protocol. I have a few comments and suggestions where more detail regarding SPIRIT checklist items may be useful. Numbers below refer to items on the SPIRIT checklist.

5d and 21a I couldn't find any reference to a data monitoring committee or other committees overseeing the trial on the page noted or using a keyword search of the manuscript.

11b Could you give more detail on criteria for discontinuing or modifying allocated interventions for a given trial participant?

11c Could you provide details on strategies to improve adherence to intervention protocols?

11d Are there any concomitant treatments or interventions that are specifically prohibited?

12 Is the primary outcome a final value or a change from baseline? Will it be aggregated as mean or median?

16b How will the allocation sequence be implemented eg telephone, sealed opaque envelopes?

16c Who will enrol participants and assign them to their interventions?

22 Will adverse events be reported and if so, to whom?

23 Could you give more detail on processes for auditing trial conduct?

25 How will important protocol modifications be communicated to interested parties?

26a Who is responsible for obtaining informed consent?

27 Can you give more detail on how patient confidentiality will be maintained?

Publons Reviewer Recognition. Springer Nature can send verification of this review directly to Publons (a subsidiary of Clarivate Analytics). If you would like to take advantage of this service, please click on the "Yes" option below. Your name, email address, title of the reviewed manuscript, name of the journal, and date of your review submission (the "Review Data") will then be transmitted to Publons after the final decision on the manuscript has been made. If you

have already registered at Publons, they will notify you of the receipt of this review and update your profile as per your settings and their policy. If you are not registered with Publons, you will receive an email from them asking you to register in order for them to be able to recognize your review on your new profile page. Publons may use the Review Data to generate derivative metadata for the benefit of Publons and you as a reviewer, carefully considering the sensitivity of such information. For example, Publons may verify your record as a reviewer by updating your profile published on its webservice if you have registered for such service or help editors to identify candidate reviewers. Please find the details of processing in Publons' privacy policy https://publons.com/about/terms

-Yes

Level of interest

Please indicate how interesting you found the manuscript:

An article of importance in its field

Quality of written English

Please indicate the quality of language in the manuscript:

-Acceptable

Quality of figures

All images and figures within the manuscript should be genuine i.e. without evidence of manipulation. No specific feature within an image may be enhanced, obscured, moved, removed, or introduced. If you have concerns about the veracity of the figures you should choose the first option below.

-Acceptable

Statistical review

Is it essential that this manuscript is seen by an expert statistician? If so, please give your reasons in your report.

-Yes, but I do not feel adequately qualified to assess the statistics.

Declaration of competing interests

Please complete a declaration of competing interests, considering the following questions:

1. Have you in the past five years received reimbursements, fees, funding, or salary from an

organisation that may in any way gain or lose financially from the publication of this manuscript, either now or in the future?

2. Do you h hold any stocks or shares in an organisation that may in any way gain or lose financially from the publication of this manuscript, either now or in the future?

3. Do you hold or are you currently applying for any patents relating to the content of the manuscript?

4. Have you received reimbursements, fees, funding, or salary from an organization that holds or has applied for patents relating to the content of the manuscript?

5. Do you have any other financial competing interests?

6. Do you have any non-financial competing interests in relation to this paper?

If you can answer no to all of the above, write 'I declare that I have no competing interests' below. If your reply is yes to any, please give details below.

-I declare that I have no competing interests

I agree to the open peer review policy of the journal. I understand that my name will be included on my report to the authors and, if the manuscript is accepted for publication, my named report including any attachments I upload will be posted on the website along with the authors' responses. I agree for my report to be made available under an Open Access Creative Commons CC-BY license (http://creativecommons.org/licenses/by/4.0/). I understand that any comments which I do not wish to be included in my named report can be included as confidential comments to the editors, which will not be published.

-I agree to the open peer review policy of the journal

Were you mentored through this peer review?

No
